# Transportation as a barrier to colorectal cancer care

**DOI:** 10.1186/s12913-021-06339-x

**Published:** 2021-04-13

**Authors:** Shelley A. Jazowski, Isabelle P. Sico, Jennifer H. Lindquist, Valerie A. Smith, Hayden B. Bosworth, Susanne Danus, Dawn Provenzale, Michael J. Kelley, Leah L. Zullig

**Affiliations:** 1 Department of Population Health Sciences, Duke University School of Medicine, Durham, NC 27701 USA; 2 Department of Health Policy and Management, University of North Carolina at Chapel Hill, Chapel Hill, NC USA; 3 Center of Innovation to Accelerate Discovery and Practice Transformation, Durham Veterans Affairs Health Care System, Durham, NC USA; 4 Division of General Internal Medicine, Duke University, Durham, NC USA; 5 School of Nursing, Duke University, Durham, NC USA; 6 Department of Psychiatry and Behavioral Sciences, Duke University, Durham, NC USA; 7 Department of Medicine, Division of Gastroenterology, Duke University School of Medicine, Durham, NC USA; 8 Cooperative Studies Program Epidemiology Center, Durham, NC USA; 9 Department of Veterans Affairs, Specialty Care Services, Washington, DC USA; 10 Durham Veterans Affairs Health Care System, Durham, NC USA; 11 Division of Medical Oncology, Duke University School of Medicine, Durham, NC USA

**Keywords:** Access to care, Colorectal cancer, Life chaos, Transportation, Travel distance, Veterans Affairs

## Abstract

**Background:**

Transportation barriers limit access to cancer care services and contribute to suboptimal clinical outcomes. Our objectives were to describe the frequency of Veterans reporting and the factors associated with transportation barriers to or from colorectal cancer (CRC) care visits.

**Methods:**

Between November 2015 and September 2016, Veterans with incident stage I, II, or III CRC completed a mailed survey to assess perceived barriers to recommended care. Participants who reported difficulty with transportation to or from CRC care appointments were categorized as experiencing transportation barriers. We assessed pairwise correlations between transportation barriers, transportation-related factors (e.g., mode of travel), and chaotic lifestyle (e.g., predictability of schedules), and used logistic regression to examine the association between the reporting of transportation difficulties, distance traveled to the nearest Veterans Affairs (VA) facility, and life chaos.

**Results:**

Of the 115 Veterans included in this analysis, 18% reported experiencing transportation barriers. Distance to the VA was not strongly correlated with the reporting of transportation barriers (Spearman’s ρ = 0.12, *p* = 0.19), but chaotic lifestyle was both positively and significantly correlated with experiencing transportation barriers (Spearman’s ρ = 0.22, *p* = 0.02). Results from the logistic regression model modestly supported the findings from the pairwise correlations, but were not statistically significant.

**Conclusions:**

Transportation is an important barrier to or from CRC care visits, especially among Veterans who experience greater life chaos. Identifying Veterans who experience chaotic lifestyles would allow for timely engagement in behavioral interventions (e.g., organizational skills training) and with support services (e.g., patient navigation).

## Background

Transportation barriers, including availability and cost of transportation and distance and time traveled to care [1], limit access to necessary healthcare services and contribute to suboptimal clinical outcomes across the cancer care continuum [2, 3]. For patients with colorectal cancer (CRC), transportation barriers have been associated with diagnoses of advanced disease [2–4] and also resulted in the decreased likelihood of receiving or adhering with recommended treatment and specialist care [2, 3, 5, 6]. In turn, non-adherence with CRC care plans – treatment, surveillance, and survivorship – has contributed to shortened survival and impaired quality of life [7, 8].

Transportation barriers and subsequent health consequences disproportionately affect older adults [6–11], patients living in rural areas [2, 4, 5, 9–11], persons of color [8, 11, 12], those of lower socioeconomic status [5–7, 10, 11], and individuals with limited social support [6, 10, 12]. U.S. Veterans are more likely to be older when diagnosed with CRC [11, 13, 14], more often to reside in rural counties [11], and have lower rates of employment [15, 16], college education [16], and income [11, 15, 16] than the general population. Despite the increased risk for experiencing transportation difficulties to or from care, only a few studies have evaluated the relationship between known patient-level factors and transportation barriers among Veterans.

Veterans Health Administration (VHA) research has supported findings related to the demographic (e.g., older age, persons of color) [17, 18], geographic (e.g., rurality) [18, 19], and lifestyle characteristics (e.g., inadequate social support) [11] associated with transportation barriers, reduced access to care, and unmet medical need among privately and publicly insured individuals [11, 17–20]. However, a majority of these studies did not focus on Veterans with CRC [17–20] or were not designed to or inconsistently measured difficulties with transportation [11, 17, 20]. Questions regarding the frequency of and which transportation-related factors (e.g., distance to care, mode of travel) are associated with self-reported transportation barriers among Veterans with CRC remain.

To address these gaps in knowledge, the objectives of this study were to determine how widespread transportation barriers were among Veterans with CRC and to assess the patient-level and transportation-related factors associated with the reporting of transportation difficulties.

## Methods

### Participants

**Fig. 1:**
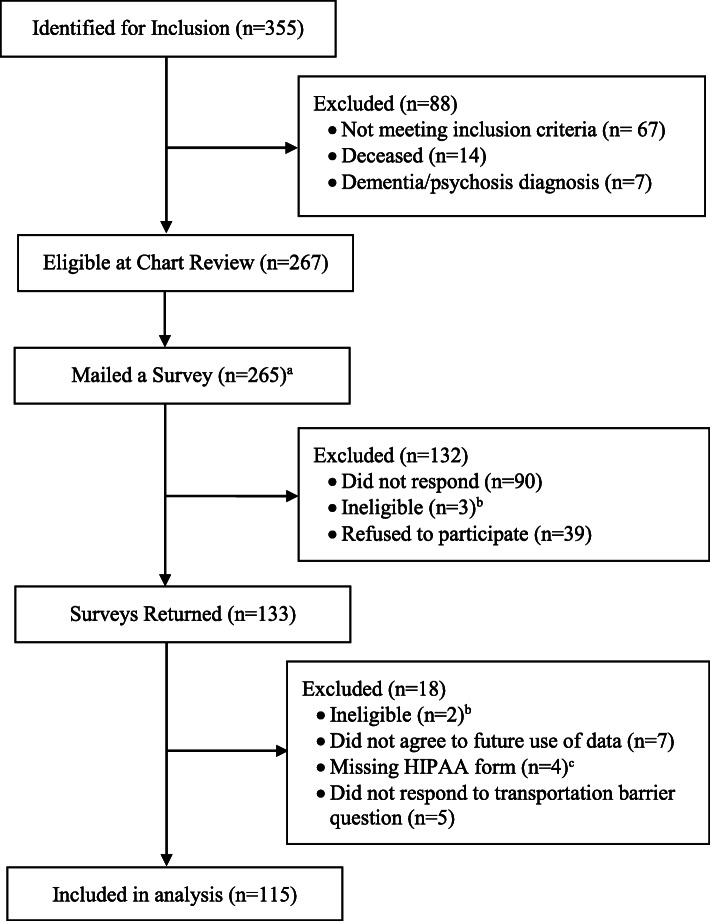
Study Participant Identification. Abbreviations: HIPAA, Health Insurance Portability and Accountability Act. ^a^ Two surveys were not mailed to Veterans because at the time of mailing these individuals were deemed ineligible since they did not meet inclusion criteria, had a dementia or psychosis diagnosis, or were deceased. ^b^ Patients were deemed ineligible because they did not meet inclusion criteria, had a dementia or psychosis diagnosis, or were deceased. ^c^ Study participants were required to complete and sign a HIPAA authorization form. Participants whose forms were not returned, incomplete, and/or lacked a signature were excluded from analysis

We used the Veterans Affairs (VA) Cancer Cube, which contains diagnostic, clinical, and treatment information about suspected cancer cases [21, 22], to identify eligible participants. We included Veterans who (1) had incident stage I, II, or III colorectal cancer, (2) were diagnosed between October 2007 and December 2015 at VA Medical Centers (VAMCs) in North Carolina or Virginia, and (3) had a valid home mailing address and telephone number. Following initial identification of 355 Veterans, we reviewed electronic health record (EHR) data to confirm eligibility criteria and vital status (Fig. 1).

### Data collection

The Colorectal Cancer Patient Adherence to Survivorship Treatment (COAST) survey assessed perceived barriers to, and adherence with, recommended care [21]. Between October 2015 and September 2016, information regarding informed consent, self-administered surveys, and non-monetary incentives (cancer survivorship pins) were mailed to 265 eligible Veterans. Approximately 2 weeks after the initial mailing, Veterans were called if surveys were not returned (to verify mailing addresses) or if information was incomplete (to collect missing survey responses). A total of 133 Veterans returned surveys, yielding an overall response rate of 50.2%.

### Ethical considerations

This study was approved by the Durham VA Health Care System Institutional Review Board. All participants included in this analysis provided implied consent by returning a completed survey and completed a Health Insurance Portability and Accountability Act (HIPAA) authorization form.

### Measures

#### Transportation barriers

The primary outcome was reporting difficulty with transportation to or from CRC treatment or a related appointment. Veterans who responded “Sometimes”, “Often”, or “Always” were categorized as having experienced transportation barriers, while those that selected “Never” were classified as having no transportation barriers [11]. Consistent with prior studies that have assessed transportation barriers [11] and cross-sectional surveys [23–25], Veterans who did not respond or reported “Don’t know” were excluded from analysis (*n* = 5).

#### Demographics

Self-reported demographics included age, sex, race, education, employment, and marital status. Veterans were categorized as either 40 to 65 years of age or 66 years or older at time of survey completion based on their year of birth and date of survey completion. Due to a limited number of racial and ethnic minority respondents (*n* = 38), we classified race as white or persons of color (American Indian or Alaska Native, Asian, black or African American, Native Hawaiian or Pacific Islander, multiracial). In addition, Veterans who reported full- or part-time employment were categorized as working. Veterans’ marital status was classified as married (married or living together), divorced or separated, widowed, or single (single or never married).

#### Transportation-related factors

We measured location of CRC care services, distance to the nearest VA hospital or clinic, convenience of the nearest VA location, and the most common mode of travel to the VA. The VA was considered the primary treatment location for Veterans who reported receiving “All”, “More than half”, or “About half” of their CRC care in VA hospitals or clinics, whereas a non-VA facility was deemed the major source of services for those receiving “Less than half” of their CRC care at the VA. Since travel distances exceeding 20 miles have been associated with delays in and/or reduced receipt of cancer care [26, 27], we categorized distance to the nearest VA as either less than or equal to 20 miles (respondents selected “0 to 20 miles”) or greater than 20 miles (Veterans selected “21 to 40 miles”, “41 to 60 miles”, “61 to 80 miles”, “81 to 100 miles” or “101 miles or more”).

#### Chaotic lifestyle

Chaotic lifestyle and environment (also referred to as life chaos) encompasses individuals’ ability to organize and anticipate future events, as well as the consistency and predictability of their daily schedules [28, 29]. Chaotic lifestyle has been associated with reduced engagement with the healthcare system [30] and non-adherence with treatment plans [28, 29], and thus, may also affect a patient’s ability to adequately plan for transportation to or from CRC care appointments. To assess Veterans’ perceived life chaos, we used the six validated measures of the Confusion, Hubbub, and Order Scale (CHAOS): “My life is organized” (reverse coded); “My life is unstable”; “My routine is the same from week to week” (reverse coded); “My daily activities from week to week are unpredictable”; “Keeping a schedule is difficult for me”; and “I do not like to make appointments too far in advance because I do not know what might come up” [30]. The CHAOS scale was scored by summing 5-point Likert scale responses ranging from “Definitely true” to “Definitely false”, with higher scores signifying a chaotic lifestyle (scores range from 6 to 30) [28–30]. We considered a CHAOS score of 16 or higher as an indication of a more chaotic lifestyle [31].

### Statistical analysis

We conducted statistical analysis in SAS version 9.4 (SAS Institute, Cary, NC). We examined differences in demographic, lifestyle, and transportation-related characteristics between Veterans who did and did not report transportation barriers using chi-square or Fisher’s exact tests. We also assessed pairwise correlations between transportation barriers, transportation-related factors, and chaotic lifestyle. Lastly, we used logistic regression to evaluate the association between the reporting of transportation barriers, distance traveled to the VA, and chaotic lifestyle.

## Results

### COAST respondent characteristics

Of the 115 survey respondents that were included in this analysis (Fig. 1), a majority were aged 66 years or older (72%), were married (59%), and had a high school diploma or higher education (87%) (Table 1). In addition, most Veterans reported primarily receiving CRC care at a VA hospital or clinic (94%), living more than 20 miles from the nearest VA (71%), and driving themselves to and from the VA for care (58%). A majority of Veterans experienced a less chaotic lifestyle (57%).

**Table 1:** COAST Respondent Characteristics

Characteristic	All Respondents (***n*** = 115)	Reported Transportation Barriers (***n*** = 21)	Reported No Transportation Barriers (***n*** = 94)	***P***-value
**Demographics (N, %)**				
Age at survey completion				
40–65	32 (28)	11 (52)	21 (22)	0.006
≥ 66	83 (72)	10 (48)	73 (78)	
Sex				
Male	109 (95)	19 (90)	90 (96)	0.301
Female	6 (5)	2 (10)	4 (4)	
Race^a^				
White	74 (64)	13 (62)	61 (65)	0.700
Persons of color	38 (33)	7 (33)	31 (33)	
Employment^a^				
Working	28 (24)	1 (5)	27 (29)	0.060
Not working	85 (74)	20 (95)	65 (69)	
Education^a^				
Less than high school	14 (12)	1 (5)	13 (14)	0.559
High school graduate or higher education	100 (87)	20 (95)	80 (85)	
Marital status				
Married	68 (59)	8 (38)	60 (64)	0.005
Divorced/separated	29 (25)	12 (57)	17 (18)	
Widowed	13 (11)	1 (5)	12 (13)	
Single	5 (4)	0 (0)	5 (5)	
**Transportation-related Factors (N, %)**				
CRC care location^a^				
VA hospital/clinic	108 (94)	19 (90)	89 (95)	0.488
Non-VA health center	5 (4)	2 (10)	3 (3)	
Distance to the VA				
≤ 20 miles	33 (29)	4 (19)	29 (31)	0.280
> 20 miles	82 (71)	17 (81)	65 (69)	
Convenience of the VA^a^				
Convenient	96 (83)	15 (71)	81 (86)	0.143
Not convenient	17 (15)	6 (29)	11 (12)	
Mode of travel				
Drive self	67 (58)	8 (38)	59 (63)	0.098
Rely on friends/family	44 (38)	12 (57)	32 (34)	
Public transportation	2 (2)	1 (5)	1 (1)	
Other method	2 (2)	0 (0)	2 (2)	
**Lifestyle Factors (N,%)**				
Chaotic lifestyle^a^				
Less chaotic	65 (57)	9 (43)	56 (60)	0.245
More chaotic	47 (41)	12 (57)	35 (37)	

Abbreviations: *COAST* Colorectal Cancer Patient Adherence to Survivorship Treatment, *CRC* colorectal cancer, *VA* Veterans Affairs

^a^Participants with missing data, which is defined as not responding or selecting “Prefer not to answer” or “Don’t know”, are not included in the table. Race was missing for 1 participant reporting barriers and for 2 participants reporting no barriers; employment was missing for 2 participants reporting no barriers; education was missing for 1 participant reporting no barriers; location of cancer care was missing for 2 participants reporting no barriers; convenience of VA location was missing for 2 participants reporting no barriers; and chaotic lifestyle was missing for 3 participants reporting no barriers

Approximately 18% (*n* = 21) of Veterans reported experiencing transportation barriers to or from CRC care appointments. Demographics, transportation-related factors, and lifestyle characteristics were similar between Veterans who did and did not report transportation barriers, although Veterans experiencing difficulties with transportation were more likely to be younger (52% vs. 22%, *p* = 0.006) and less likely to be married (38% vs. 64%, *p* = 0.005) than those without barriers.

### Correlation between transportation barriers, transportation-related factors, and life chaos

**Fig. 2:**
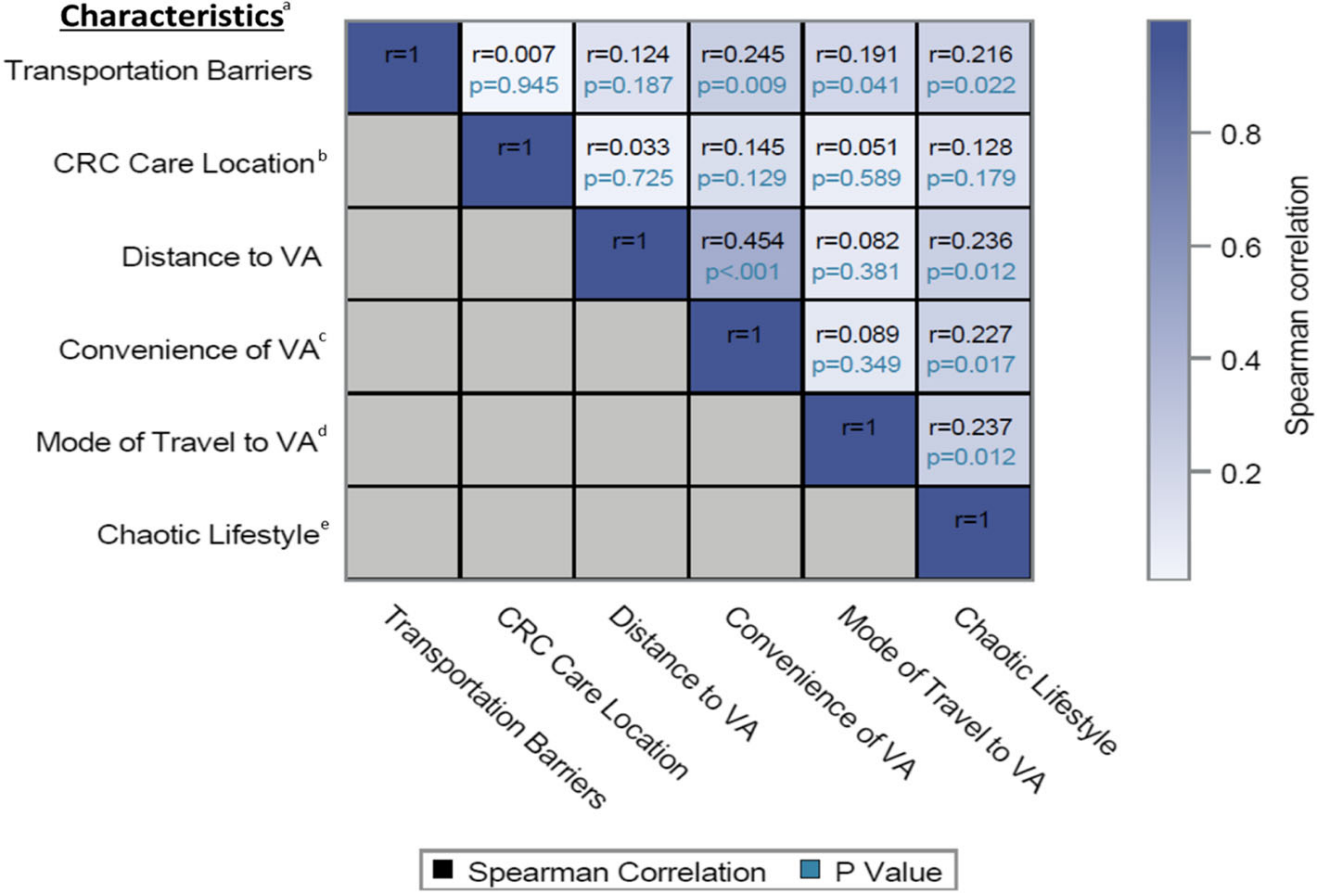
Relationship Between Transportation-related Factors and Chaotic Lifestyle. Abbreviations: CRC, colorectal cancer care; VA, Veterans Affairs. ^a^ SAS was used to calculate Spearman’s correlation coefficients and to create the correlation heatmap. Variables were continuous (chaotic lifestyle) or categorical (transportation barriers: “Always”, “Often”, “Sometimes”, “Never”; distance to the VA: “0 to 20 miles”, “21 to 40 miles”, “41 to 60 miles”, “61 to 80 miles”, “81 to 100 miles”, “101 miles or more”; use of the VA for colorectal cancer care: “All of your cancer care”, “More than half of your cancer care”, “About half of your cancer care”, “Less than half of your cancer care”; convenience of VA: “Very convenient”, “Somewhat convenient”, “Somewhat inconvenient”, “Very convenient”; mode of travel to the VA: “Drive myself’, “Friend or family drives me”, “Health aide”, “Disabled American Veterans (DAV) or other shuttle service”, “Public transportation”, “Another source”). ^b^ Participants who did not respond or responded “Don’t know” were excluded from analysis (*n* = 2). ^c^ Participants who did not respond or responded “Don’t know” were excluded from analysis (*n =* 2). ^d^ Mode of travel to the VA is ordered based on decreasing travel independence. Veterans who selected (1) “Drive myself” have access to a vehicle and are in control of traveling to or from the VA as needed; (2) “Friend or family drives me” face some limitations due to others’ availability but are able to tailor transportation to or from the VA according to their needs; (3) “Public transportation” are further limited by fixed travel schedules and routes to or from the VA; or (4) “Another source” are assumed to be the most restricted in terms of travel independence (respondents did not select “Health aide” or “DAV or other shuttle service”). ^e^ Participants who did not respond or had missing values were excluded from analysis (*n* = 3)

Distance to the VA was not strongly correlated with reporting transportation barriers to or from CRC care visits (Spearman’s ρ = 0.124, *p* = 0.187), but mode of travel was significantly correlated with reporting transportation barriers (Spearman’s ρ = 0.191, *p* = 0.041) (Fig. 2). Convenience of the VA and chaotic lifestyle were both positively and significantly correlated with experiencing transportation barriers (Spearman’s ρ = 0.245, *p* = 0.009 and ρ = 0.216, *p* = 0.022, respectively) and distance to the nearest VA (Spearman’s ρ = 0.454, *p* < 0.001 and ρ = 0.236, *p* = 0.012).

### Factors associated with reporting transportation barriers

Results from the logistic regression model modestly supported the findings from the pairwise correlations, but were not statistically significant. Findings suggest that Veterans living further from the VA and experiencing greater life chaos have a higher probability of transportation difficulties than those residing within 20 miles of care and reporting a less chaotic lifestyle (predicted probability = 0.272, 95% confidence limit [CL] = 0.159, 0.425 vs. predicted probability = 0.102, 95% CL = 0.036, 0.258) (Table 2).

**Table 2:** Predicted Probabilities of Reporting Transportation Barriers

Distance to the VA	Chaotic Lifestyle	Predicted Probability (95% CL)
≤ 20 miles	Less chaotic lifestyle (score < 16)	0.102 (0.036, 0.258)
≤ 20 miles	More chaotic lifestyle (score ≥ 16)	0.184 (0.063, 0.431)
> 20 miles	Less chaotic lifestyle (score < 16)	0.158 (0.080, 0.291)
> 20 miles	More chaotic lifestyle (score ≥ 16)	0.272 (0.159, 0.425)

Abbreviations: *CL* confidence limits, *VA* Veterans Affairs

^a^We used PROC LOGISTIC in SAS to estimate the predicted probability of transportation barriers for Veterans who (1) traveled ≤20 miles to the VA and had a less chaotic lifestyle, (2) traveled ≤20 miles to the VA and had a more chaotic lifestyle, (3) traveled > 20 miles to the VA and had a less chaotic lifestyle, and (4) traveled > 20 miles to the VA and had a more chaotic lifestyle (the reference group is Veterans who reported no difficulties with transportation to or from cancer care appointments). Predicted probabilities were not statistically significant for all groups

^b^We tested goodness of fit with deviance and Pearson chi square tests, which indicated reasonable model fit

## Discussion

To our knowledge, this is one of the first studies to evaluate transportation-related factors (e.g., distance to care) and lifestyle characteristics (e.g., life chaos) associated with reporting transportation barriers among Veterans with CRC. Findings indicated that mode of travel was significantly correlated with transportation barriers to or from CRC care appointments, suggesting that Veterans who rely on family, friends, or public transportation may not have consistent access to or use of reliable transportation. Treatment side-effects (e.g., fatigue, vision impairment) [11, 32, 33], coupled with VA care requirements (e.g., an adult must attend a colonoscopy appointment) [34] increases Veterans’ need for and dependence on a stable support system for transportation to or from care. However, competing priorities – employment demands and family obligations – may limit caretakers’ ability to regularly drive a Veteran to CRC treatment or medical visits [35, 36].

Although transportation services to or from cancer-related appointments (e.g., VA, community healthcare facility, or patient advocacy programs) have eased the burden of both patients and their caregivers [36, 37], none of the study participants selected or discussed these programs in their survey responses. Veterans may be unaware of the transportation services offered by the VA or their community [38] or they may find available services to be inconvenient (e.g., infrequent pick-off and drop-off times, difficulty traveling to designated pick-up or drop-off locations) [18, 20] or infeasible for their care needs (e.g., not adequately equipped for Veterans using wheelchairs) [18]. Future studies should identify ways in which transportation programs can be aligned with patients’ needs and the circumstances in which telehealth services may replace in-person appointments [18–20].

We also found that distance to the nearest VA facility was not significantly correlated with the reporting of transportation barriers to or from CRC care appointments. This finding suggests that Veterans may have accessed community services located closer to their homes or travel distance may not be a substantial deterrent to the care and support received at VA hospitals or clinics. Some Veterans have cited the importance of the patient-provider relationship [39, 40] as a primary reason for seeking care at the VA, while others value camaraderie – specifically, the kinship in shared military service and cancer care experiences [39, 41]. Together, these factors have been associated with increased patient engagement and adherence with recommended care and improved quality of life [42–45]. Despite these advantages, there may be a threshold at which distance to a VA facility becomes inconvenient and may lead to barriers to or from CRC care.

In some cases, Veterans live more than 100 miles from the nearest VA facility and distance may become a more pronounced barrier to or from care with each CRC treatment or surveillance appointment [16, 18]. Due to the increased risk of delays in care resulting from transportation barriers, VA facilities have established cancer care navigation teams (CCNTs). CCNTs assist high-risk Veterans (those living 100 miles or more from care, receiving multi-modality treatment, or experiencing significant psychosocial barriers to care) with addressing identified barriers to cancer care [16]. For example, CCNTs manage the logistics (e.g., accommodations, transportation) for each appointment and identify other services (e.g., medication management, counseling) that meet Veterans’ medical and psychosocial needs [16]. Future research is necessary to assess the feasibility of implementing or expanding CCNTs across VA facilities, especially since patient navigation services minimize barriers to cancer care, improve the education and support of Veterans, and have the potential to ensure continuity of care across the cancer care continuum [16, 46, 47].

Finally, we noted that a chaotic lifestyle was significantly correlated with the reporting of transportation barriers to or from CRC care appointments. A chaotic lifestyle may affect Veterans’ ability to identify, schedule, and manage access to reliable transportation; however, transportation instability (e.g., public transportation delays or cancelations, inability to afford transportation expenses) may disrupt Veterans’ lives and yield a higher degree of life chaos. Future research is needed to explore this potentially endogenous relationship, as well as to discern which facet of a chaotic lifestyle is the most challenging for Veterans experiencing transportation difficulties and which transportation-related factor is driving life chaos [28]. In turn, this information could determine what type of and when an intervention (e.g., organizational skills training, telehealth services) is warranted [28].

This study had several limitations. First, a small sample size and inability to adjust for potential measured confounders (e.g., demographics) contributes to lack of statistical significance and limits the interpretation and validity of our findings. Future studies should survey a broader population of Veterans with CRC to enable a robust examination of the factors associated with the reporting of transportation barriers. Second, our study focused on a regional sample of Veterans with non-metastatic CRC who had a valid home mailing address, which further limits the generalizability of our findings. Future research is necessary to determine if the frequency of and factors associated with reporting transportation barriers differs among female Veterans and those with advanced disease. Third, based on the definition of the primary outcome, our study did not determine how the association between life chaos and transportation-related factors may have varied across the degrees (e.g., always vs. sometimes) of experiencing transportation barriers. Fourth, we measured distance to and convenience of respondents’ nearest VA location; however, we were not able to discern if this facility was the primary provider of CRC services. Fifth, similar to all surveys, our study may be subject to potential recall and response biases; however, the use of validated and previously tested measures may have minimized the effect of these biases [48]. Sixth, our response rate of 50% may have introduced selection bias as non-responders may have different sociodemographic factors, lifestyle characteristics, and transportation needs than survey respondents. Seventh, as with any observational study, unmeasured confounding could be a potential source of bias. Eighth, this study was not designed to assess the effect of transportation barriers on the use of cancer care services. Future research should not only account for how difficulties with transportation impact CRC care, but also determine whether policies aimed at expanding access (e.g., Choice Act, MISSION Act) [49, 50] have minimized the negative impact of transportation barriers. Lastly, the cross-sectional nature of this study limits our ability to establish a causal relationship between chaotic lifestyle, distance to care, and transportation barriers.

## Conclusions

Our findings suggest that transportation is an important barrier to or from CRC care visits, especially among Veterans who experience greater life chaos. Future research is needed to determine the association between perceived life chaos and receipt of CRC care (e.g., delayed therapy initiation, missed appointments). However, screening for life chaos should be integrated into routine clinical practice. Identification of Veterans who experience chaotic lifestyles would allow for timely intervention – organizational skills training, patient navigation, and/or telehealth services – which in turn, could lead to the potential modification of observed risk factors and support the continuity of care across the cancer care continuum [16, 36, 37, 46, 47].

## Data Availability

The datasets (including de-identified datasets) generated and analyzed during this study are not available publicly or upon request due to privacy and regulatory approvals.

## Abbreviations

CCNT: Cancer care navigation team
CHAOS: Confusion, Hubbub, and Order Scale
CL: Confidence limit
COAST: Colorectal Cancer Patient Adherence to Survivorship Treatment
CRC: Colorectal cancer
DAV: Disabled American Veterans
EHR: Electronic health record
HIPAA: Health Insurance Portability and Accountability Act
US: United States of America
VA: Veterans Affairs
VAMC: Veterans Affairs Medical Center
VHA: Veterans Health Administration
